# Application of preoperative NLR-based prognostic model in predicting prognosis of intrahepatic cholangiocarcinoma following radical surgery

**DOI:** 10.3389/fnut.2024.1492358

**Published:** 2024-10-30

**Authors:** Shuo Qi, Zhongzhi Ma, Lian Shen, Jun Wang, Lei Zhou, Bingzhang Tian, Changjun Liu, Kang Chen, Wei Cheng

**Affiliations:** Department of Hepatobiliary Surgery, Hunan Provincial People’s Hospital, The First Affiliated Hospital of Hunan Normal University, Changsha, China

**Keywords:** intrahepatic cholangiocarcinoma, lymphocyte count ratio, radical surgery, prognostic model, prediction

## Abstract

**Purpose:**

To investigate the application value of the neutrophil to lymphocyte count ratio (NLR) in the prognostic analysis of intrahepatic cholangiocarcinoma (ICC) after radical resection, and to offer guidance for the individualized perioperative diagnosis and treatment of ICC.

**Methods:**

The clinical data of 360 patients diagnosed with ICC following radical surgery were retrospectively analyzed. The cut-off value of NLR was calculated using the minimum *p*-value method, and then divided into High-NLR (H-NLR) group and Low-NLR (L-NLR) group according to the NLR cut-off value. The prognostic value of NLR in ICC was analyzed. Subsequently, the patients were divided into the hepatolithiasis-related intrahepatic cholangiocarcinoma (HICC) group and the non-hepatolithiasis-related intrahepatic cholangiocarcinoma (NHICC) group based on whether they combined with hepatolithiasis. Multiple regression models were constructed based on NLR and clinicopathological indicators to verify the application value of prognostic models in the survival and recurrence of ICC patients after radical surgery.

**Results:**

The cut-off value of NLR was 2.36, and the survival analysis disclosed that overall ICC patients with NLR ≥ 2.36 manifested a poor 5-year survival rate and a higher tumor recurrence rate (*p* < 0.001). In the HICC group, patients with H-NLR presented a poor 5-year survival rate and a higher tumor recurrence rate compared with L-NLR (*p* < 0.001). The NLR-based survival/recurrence prediction models in the HICC group demonstrated excellent predictive capacity (H-L test: 0.359/0.680, AUC: 0.764/0.791). In the NHICC group, patients with H-NLR exhibited a poor 5-year survival rate compared with L-NLR (*p* < 0.001), yet there was no significant difference in tumor recurrence between the two groups (*p* = 0.071). The NLR-based survival prediction model in the NHICC group demonstrated acceptable predictive ability (H-L test: 0.268, AUC: 0.729), while the NLR-based recurrence prediction model did not show an effective predictive ability (H-L test: 0.01, AUC: 0.649).

**Conclusion:**

NLR is an independent risk factor influencing postoperative survival and recurrence in ICC patients, particularly in HICC patients. Preoperative NLR ≥ 2.36 suggests that patients might have a poor prognosis. The survival and recurrence prediction model constructed based on NLR and other clinical indicators demonstrates good prediction accuracy and can effectively predict the risk of postoperative adverse prognosis in patients with HICC. This study offers a novel idea for the clinical treatment of HICC patients.

## Introduction

Intrahepatic cholangiocarcinoma (ICC) is a lethal malignancy and ranks as the second most common primary liver cancer, comprising 20% of all liver malignancies and 3% of all gastrointestinal malignancies (1, 2). Over the past four decades, the incidence of ICC has surged by over 140% (3). Despite current studies revealing an inadequate understanding of its etiology, risk factors with varying degrees of susceptibility are increasingly being identified. The most closely correlated risk factors to ICC include bile duct stones, choledochal cysts, cirrhosis cholangitis, chronic biliary tract diseases, viral hepatitis (specifically hepatitis B virus and hepatitis C virus), liver fluke infestations such as *Clonorchis sinensis* (4–6).

Surgical resection stands out as the most effective treatment method for ICC (7). Nonetheless, only 35% of patients are eligible for surgical resection at diagnosis; furthermore, the outcomes following surgical intervention remain suboptimal. The five-year survival rate post-R0 resection hovers around 30–40% (8). A multitude of factors impact ICC prognosis; key ones include tumor number and size, major vascular invasion, presence of extrahepatic disease, morphological type and histological grade of tumors, presence of lymph node metastasis,final resection margin residual free from any microscopic or macroscopic tumor thrombus/extension along insular structures left behind after any attempted excision within patient’s own factor like age gender performance score used in routine medical care assessment based on the ability to walk independently overall health status, surgical approach, molecular characteristics including specific gene mutations, and growth factor receptor expression levels (9–11). It is therefore imperative that we accurately evaluate the prognosis pertaining to ICC patients while developing corresponding treatment strategies (12). By considering individual differences among patients, we can adopt a multidisciplinary team-based personalized approach in order to optimize both prognosis and quality-of-life outcomes for individuals afflicted with this condition.

Previous research has demonstrated the significant role of serum inflammatory factors in ICC (13, 14). The analysis of preoperative inflammatory factors can provide valuable insights into the prognosis and progression of ICC, offering new perspectives and strategies for its diagnosis and treatment. Preoperative assessment of serum neutrophil/lymphocyte ratio (NLR), lymphocyte/monocyte ratio (LMR), platelet *NLR (systemic immuno-inflammation index, SII) is commonly utilized to evaluate the overall inflammatory status and immune function of tumor patients, and has been linked to tumor prognosis and treatment response in various malignancies such as digestive tract tumors (e.g., stomach cancer, colorectal cancer, pancreatic cancer), breast cancer, thyroid cancer, among others (15–17). Studies have also investigated the role of serum inflammatory factors in intrahepatic cholangiocarcinoma, revealing their significant involvement in the pathogenesis and progression of this disease (18–20). Understanding these factors can enhance our ability to manage ICC more effectively and offer specific directions for its treatment.

However, there have been limited studies on the role of serum inflammatory factors in ICC combined with hepatolithiasis, being a prevalent benign biliary condition in Asian countries (21). Prolonged stone stimulation and obstruction can lead to hepatolithiasis-related liver cirrhosis, which, if left untreated, may progress to biliary carcinogenesis. In light of this situation, we aim to categorize ICC into hepatolithiasis-related intrahepatic cholangiocarcinoma (HICC) and non-hepatolithiasis-related intrahepatic cholangiocarcinoma (NHICC) in order to investigate the role of inflammatory factors in these conditions. Our objective is to comprehend the prognostic significance of preoperative serum NLR, LMR, and SII in the resection of ICC and subsequently explore the impact of these inflammatory indicators on its prognosis based on our findings.

## Patients and methods

### Study population

This retrospective study collected patients from the Department of Hepatobiliary Surgery at Hunan Provincial People’s Hospital (the First Affiliated Hospital of Hunan Normal University) between January 2015 and December 2021. The patients included in the study had been pathologically diagnosed with ICC and had undergone radical surgery. A total of 525 patients with ICC were screened for inclusion in the study. The research was conducted in compliance with the protocol approved by the Ethics Committee of Hunan Provincial People’s Hospital (Approval No.: [2024]-121), following the principles outlined in the Declaration of Helsinki.

All the patients encompassed in the current study fulfilled the subsequent criteria: (1) underwent R0 resection, with postoperative pathology confirming ICC; comprehensive clinical case data and follow-up data were accessible; (2) did not undergo tumor-targeted treatments such as transcatheter arterial chemoembolization (TACE), chemotherapy, radiotherapy, targeted therapy, or immunotherapy, etc.; (3) preoperative Child-Pugh score of A/B; (4) had no history of previous malignant tumors. Patients with the following attributes were excluded: (1) R1/2 excision or distant metastasis (M1); (2) postoperative pathology indicating primary liver cancer such as hepatocellular carcinoma, mixed hepatocellular carcinoma, or other types of biliary malignancy; (3) patients with infectious diseases prior to operation; (4) taking hormones, aspirin, clopidogrel, and other drugs influencing peripheral blood cell indicators before surgery; (5) combined with blood diseases or immune system disorders; (6) postoperative perioperative mortality; (7) incomplete clinical data. After screening based on the inclusion and exclusion criteria, a total of 165 patients were excluded as they did not meet the inclusion criteria, and 360 patients with ICC were selected for inclusion in this study.

### Analysis of indicators

**Figure 1:**
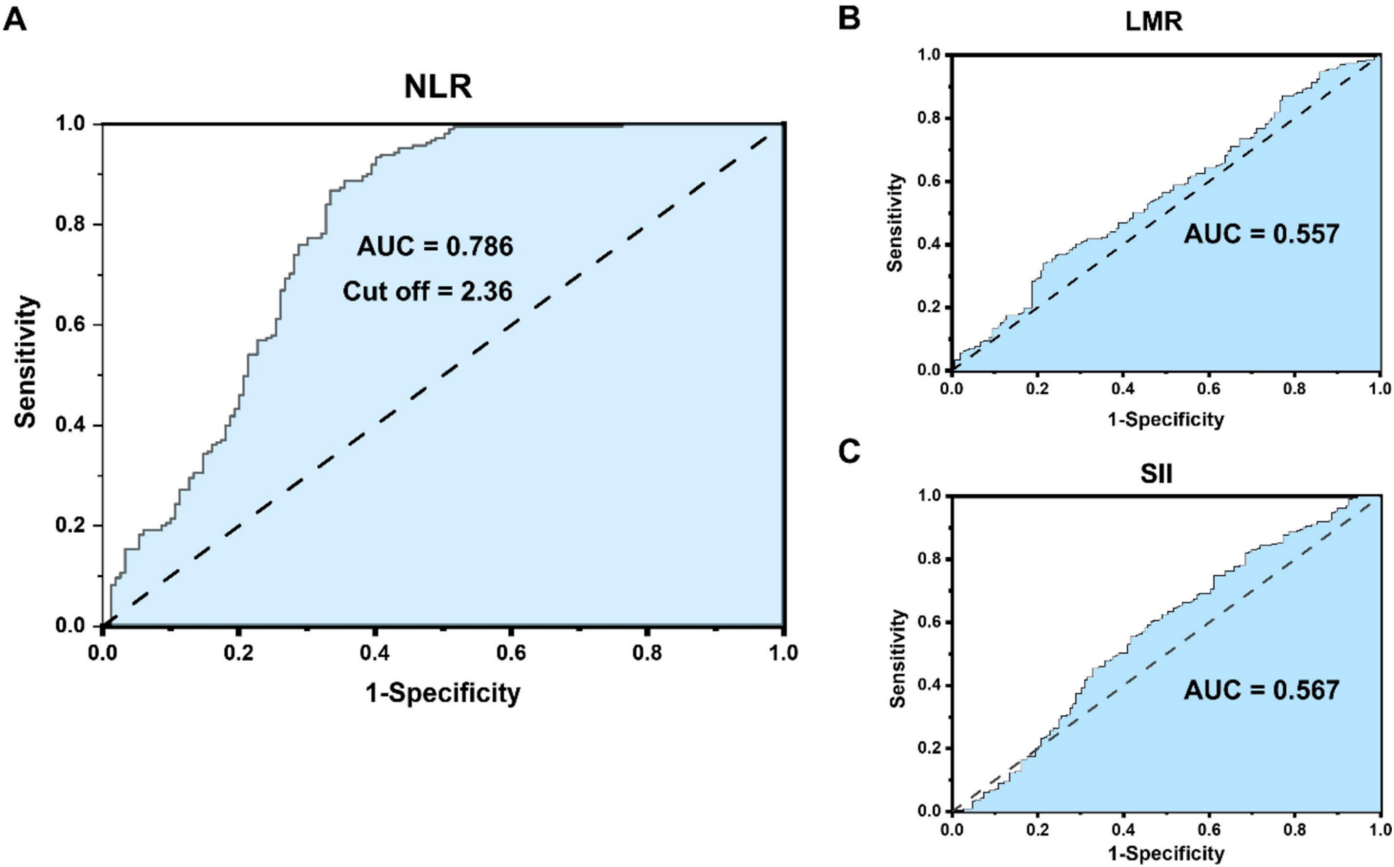
**(A)** NLR receiver operating characteristic (ROC) curve correlation and cutoff value with the prognosis of intrahepatic cholangiocarcinoma (ICC) patients. **(B,C)** LMR and SII ROC curve correlation with the prognosis of ICC patients.

The preoperative NLR (ratio of neutrophils to lymphocytes), LMP (ratio of lymphocytes to monocytes), and SII (platelet * NLR) were all derived from the preoperative case data of patients. The cut-off values of LMR, SII and NLR were, respectively, 3.71, 730.14 and 2.36, employing the maximum Youden index method. As depicted in Figure 1, ROC curves were constructed to assess the correlation between three preoperative serum inflammatory indicators (LMR, SII, NLR) and the postoperative survival of patients, and the AUC values were 0.557, 0.567, and 0.786, respectively. The AUCs of both LMR and SII were less than 0.6, indicating that there was no significant correlation between them and the prognosis of ICC patients. Hence, they are not important indices in this study. The AUC of NLR is 0.786, and the cut-off value is 2.36, which is included in the significant indices of this study. The preoperative maximum tumor diameter (MTD), hepatolithiasis, vascular invasion, local invasion, and nerve invasion were all obtained from the preoperative CT/MR Imaging data. The TNM staging was in accordance with the AJCC eighth edition installment (22). Postoperative complications were graded in accordance with the Clavien-Dindo complication criteria (23).

### Follow-up assessments

In this study, outpatient review and telephone contact were employed to carry out follow-up and follow-up investigation. The follow-up period extended to June 2024. The principal contents of follow-up observation encompassed follow-up treatment, recurrence time, and survival time of patients. Regular postoperative follow-up should be conducted, specifically every 3 to 6 months. Postoperative follow-up should incorporate: imaging examinations, such as CT, MRI, etc., to assess the local control of the tumor after surgery and detect possible recurrence or metastasis; Tumor markers, such as AFP, CEA, CA19-9, etc., to evaluate tumor activity status and potential risk of recurrence. Overall survival (OS) was defined as from the day of surgery until the time of death or the time of the last follow-up, and patients who remained alive at the last follow-up were recorded as censored data.

### Statistical analysis

The primary endpoints of this study encompassed the overall survival (OS) and recurrence rates at 1, 3, and 5 years subsequent to surgery. The postoperative survival time pertains to the duration from the date of surgery to the patient’s decease or the last follow-up, while the postoperative recurrence time refers to the period from the date of surgery to the emergence of distant metastases in other bodily regions or the last follow-up. All measurement data were presented as *mean ± standard deviation*, and the disparities of measurement data were compared by means of independent sample *t* test or *Mann–Whitney U* test. The count data were expressed as n (%), and the count data were compared via *Chi-square* test or *Fisher exact* test. The difference in tumor survival and recurrence was compared through *log-rank* test. All binary variables were classified by referring to international authoritative literature classification, clinical normal limit and cut-off values based on the best Youden index. The survival and recurrence curves were delineated by the *Kaplan–Meier curve* method. A single factor logistic regression model was utilized to estimate the probability ratio and 95% confidence interval for various variables, and variables with statistical differences in the single factor logistic regression model were incorporated into multivariate analysis. *Multivariate analysis* was used to get the prediction model, and *Hosmer-Lemeshow* test was used to check the fit degree of the model. SPSS 25.0 statistical software was employed for analysis, and *p* value less than 0.05 was regarded as statistically significant.

## Results

### Study design

**Figure 2:**
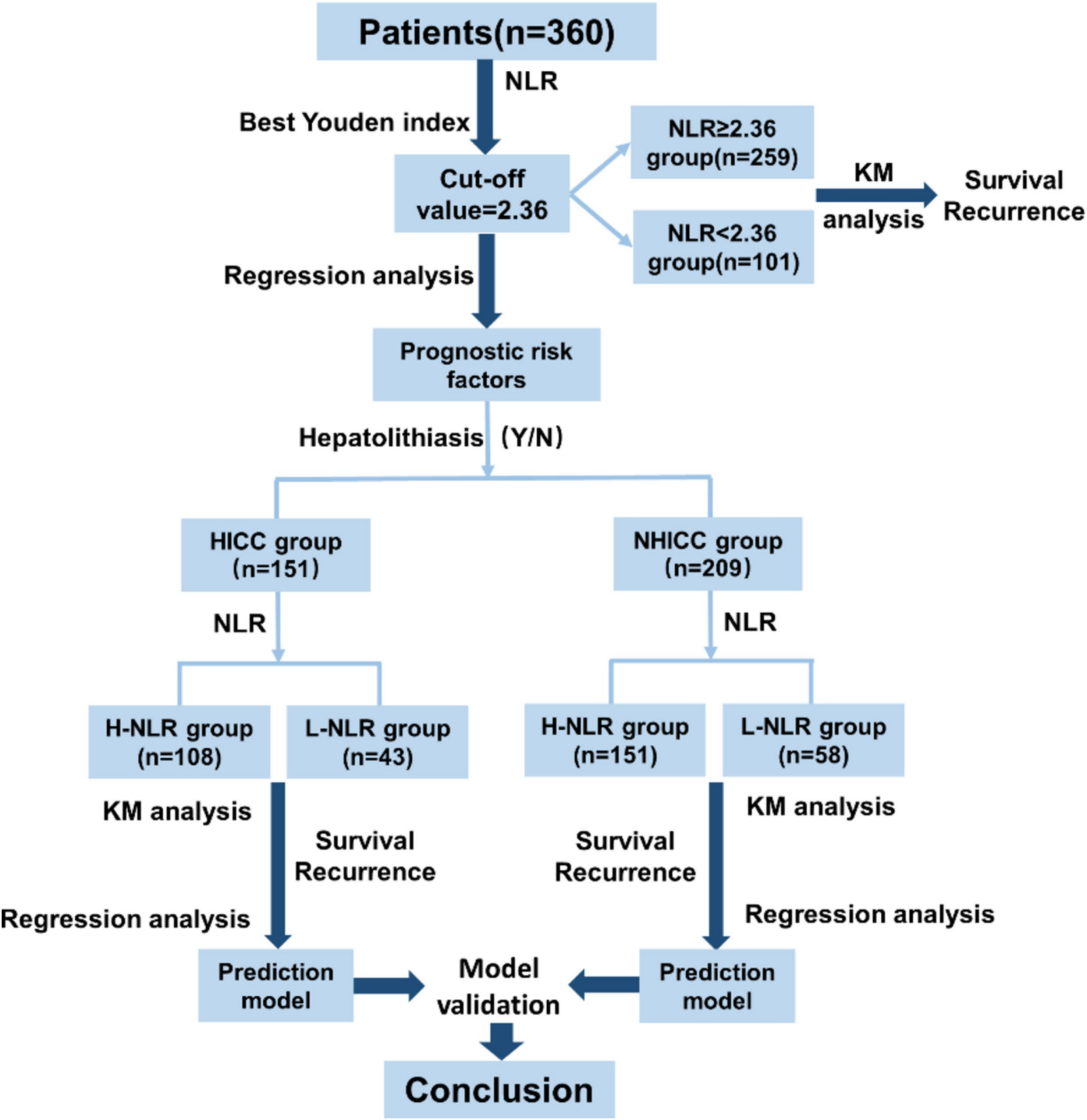
Flowchart of manuscript design.

The flowchart of the manuscript design is presented in Figure 2. In this study, a total of 360 ICC patients were recruited. Based on the best Youden index of NLR, all ICC patients were categorized into the NLR < 2.36 group and the NLR ≥ 2.36 group, and subsequently, the prognostic analysis of the two groups was compared. Then, all ICC patients were divided into the HICC group and the NHICC group depending on whether they combined with hepatolithiasis. According to the value of NLR = 2.36, the two groups were further classified into the low-NLR group and the high-NLR group, and the prognosis in each group was compared. Subsequently, the prognosis prediction model based on NLR in each group was constructed.

### Patients characteristics

The clinical characteristics of ICC patients in the NLR < 2.36 group and the NLR ≥ 2.36 group are presented in Table 1. Among the two cohorts, factors such as age, ALB, A/G, ALT, AST, ALP, GGT, PT, AFP, CA199, CA125, MTD, gender, hepatolithiasis, HBV carrier, Child-Pugh (A/B), vascular invasion, local invasion, poorly differentiation (PD), TNM staging, and complications grade between the two groups demonstrated no significant difference (*p* > 0.05), while factors including TBIL, CEA, LMR, SII, NLR, nerve invasion, and blood transfusion between the two groups showed a statistically significant difference (*p* < 0.05).

**Table 1:** Clinical characteristics of ICC patients.

Variate	Total	NLR < 2.36	NLR ≥ 2.36	*p*- value
	(*n* = 360)	(*n* = 101)	(*n* = 259)	
Age (y)	58.6 ± 9.3	57.8 ± 9.3	58.9 ± 9.3	0.681
TBIL (μmol/L)	57.2 ± 31.9	37.2 ± 24.7	63.2 ± 34.8	0.003
ALB (g/L)	39.4 ± 5.9	39.9 ± 5.9	40.2 ± 16.2	0.337
A/G	1.6 ± 0.4	1.6 ± 0.3	1.7 ± 2.8	0.429
ALT (U/L)	74.5 ± 52.6	73.8 ± 52.1	79.2 ± 53.0	0.723
AST (U/L)	61.0 ± 48.2	60.2 ± 50.3	61.1 ± 41.7	0.435
ALP (U/L)	197.9 ± 175.5	195.9 ± 185.1	202.9 ± 167.0	0.845
GGT (U/L)	270.0 ± 212.6	232.0 ± 192.2	280.8 ± 220.5	0.187
PT (s)	11.2 ± 1.4	11.1 ± 1.4	11.2 ± 1.4	0.393
AFP (ng/mL)	15.4 ± 77.3	11.9 ± 35.3	16.7 ± 88.4	0.453
CEA (ng/mL)	27.2 ± 7.8	10.3 ± 5.7	43.9 ± 12.4	0.014
CA199 (U/mL)	425.0 ± 233.5	324.1 ± 186.8	455.8 ± 248.6	0.213
CA125 (U/mL)	90.1 ± 50.2	91.1 ± 54.7	88.5 ± 48.1	0.207
LMR	3.0 ± 1.5	3.8 ± 1.7	2.6 ± 1.3	<0.001
SII	999.2 ± 944.7	573.0 ± 559.3	1095.0 ± 1086.9	<0.001
NLR	3.9 ± 1.5	1.5 ± 0.6	4.9 ± 2.4	<0.001
MTD				
>5 cm	168 (45.7%)	47 (46.5%)	121 (46.7%)	0.535
<5 cm	192 (53.3%)	54 (53.5%)	138 (53.3%)	
Gender				
Male	176 (48.9%)	49 (48.5%)	127 (49.0%)	0.512
Female	184 (51.1%)	52 (51.5%)	132 (51.0%)	
Hepatolithiasis				
Yes	151 (41.9%)	36 (35.6%)	115 (44.4%)	0.081
No	209 (58.1%)	65 (64.4%)	144 (55.6%)	
HBV carrier				
Yes	65 (18.1%)	21 (20.8%)	44 (17.0%)	0.243
No	295 (81.9%)	80 (79.2%)	215 (83.0%)	
Child-Pugh				
A	329 (91.4%)	96 (95.0%)	233 (90.0%)	0.086
B	31 (8.6%)	5 (5.0%)	26 (10.0%)	
Vascular invasion				
Yes	120 (33.3%)	34 (33.7%)	86 (33.2%)	0.514
No	240 (66.7%)	67 (66.3%)	173 (66.8%)	
Local invasion				
Yes	107 (29.7%)	35 (34.7%)	72 (27.8%)	0.125
No	253 (70.3%)	66 (65.3%)	187 (72.2%)	
Nerve invasion				
Yes	141 (39.2%)	28 (27.7%)	113 (43.6%)	0.004
No	219 (60.8%)	73 (72.3%)	146 (56.4%)	
Blood transfusion				
Yes	58 (16.1%)	8 (8.0%)	50 (19.3%)	0.005
No	302 (83.9%)	93 (92.0%)	209 (80.7%)	
Poorly differentiation				
No	182 (50.6%)	44 (43.6%)	138 (53.3%)	0.062
Yes	178 (49.4%)	57 (56.4%)	121 (46.7%)	
TNM staging				
I, II	214 (59.4%)	46 (45.5%)	100 (38.6%)	0.139
III	146 (40.6%)	55 (54.5%)	159 (61.4%)	
Complications Grade				
I, II	298 (82.8%)	83 (82.2%)	215 (83.0%)	0.481
III-V	62 (17.2%)	18 (17.8%)	44 (17.0%)	

### Comparison of overall survival and recurrence of all ICC patients

**Figure 3:**
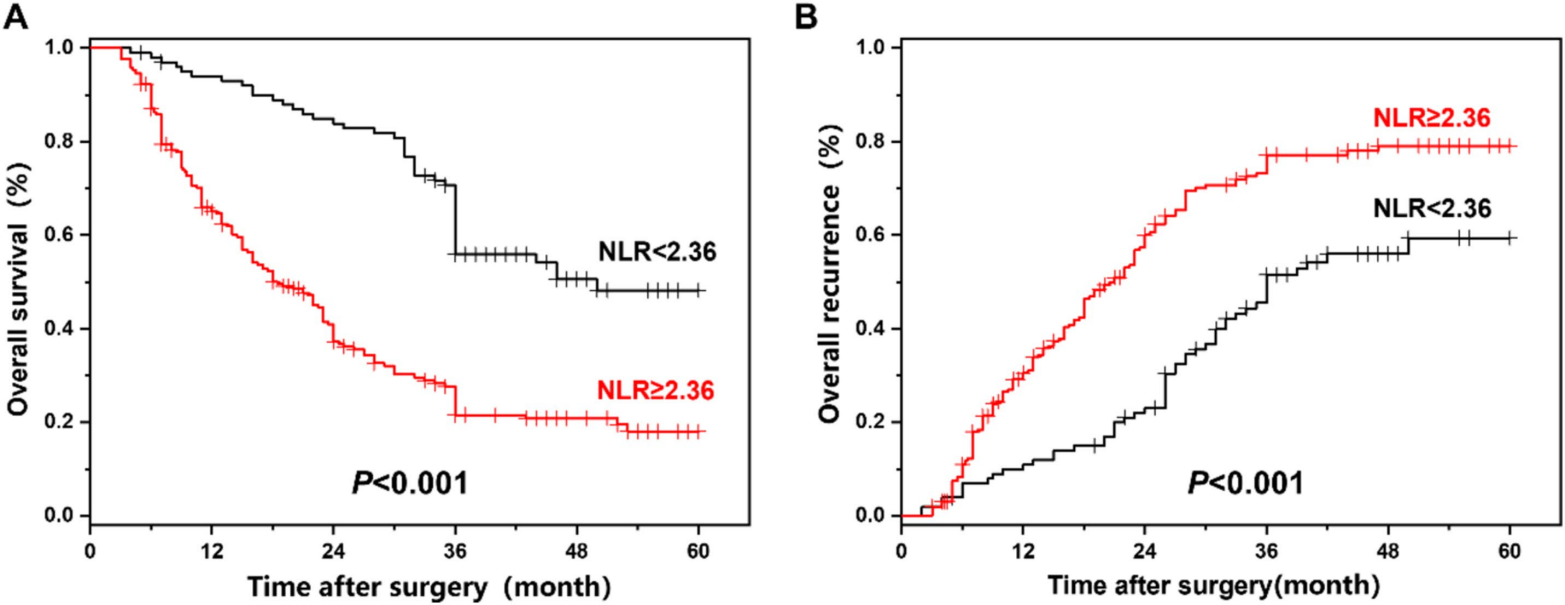
**(A,B)** The overall survival and recurrence comparison in all ICC patients between the NLR.

The comparison of overall survival and recurrence in all ICC patients between the NLR < 2.36 group and the NLR ≥ 2.36 group is presented in Figure 3. The median overall survival time of 360 ICC patients was 27 months, and the 1-year, 3-year, and 5-year overall survival rates were 74.0, 41.3, and 27.4%, respectively. Among ICC patients with NLR < 2.36, the 1-year, 3-year, and 5-year survival rates were 92.9, 70.7, and 48.2%, respectively; while among ICC patients with NLR ≥ 2.36, the 1-year, 3-year, and 5-year survival rates were 65.1, 27.7, and 18.0%, respectively, showing a statistically significant difference (*p* < 0.001). The median overall recurrence time was 24 months, and the overall recurrence rates at 1, 3, and 5 years were 24.8, 68.8, and 72.5%, respectively. Among ICC patients with NLR < 2.36, the recurrence rates at 1, 3, and 5 years were 11.1, 45.6, and 59.4%, respectively; while among ICC patients with NLR ≥ 2.36, the recurrence rates at 1, 3, and 5 years were 30.6, 73.3, and 79.1%, respectively, also showing a statistically significant difference (*p* < 0.001).

### Analyses of prognostic factors for OS and recurrence in all ICC patients

The univariable and multivariate regression analyses of the risk factors for mortality within 5 years after surgery in all ICC patients are presented in Table 2. Univariate analysis indicated that the variables related to the overall survival (OS) of ICC patients are as follows: TBIL >17.1 μmol/L, PT > 13 s, CEA > 10 ng/mL, LMR > 3.71, NLR ≥ 2.36, combined with hepatolithiasis, vascular invasion, local invasion, nerve invasion, blood transfusion, PD, and TNM stage-III. Multivariate analysis suggested that the variables associated with the OS of ICC patients are as follows: CEA > 10 ng/mL, NLR ≥ 2.36, local invasion, PD, and TNM stage-III.

**Table 2:** Univariable and multivariate regression analyses of risk factors for mortality within 5 years after surgery in ICC patients.

Variate	Univariate analysis			Multivariate analysis		
	*p*- value	HR	95%CI	*p*- value	HR	95%CI
Age > 60 y	0.455	0.851	0.577–1.300			
TBIL>17.1 μmol/L	**0.013**	1.775	1.129–2.791	0.075	0.607	0.350–1.052
ALB<35 g/L	0.589	0.875	0.539–1.420			
A/G < 1.5	0.905	0.975	0.638–1.489			
ALT>80 U/L	0.984	1.006	0.561–1.803			
AST > 80 U/L	0.345	1.380	0.707–2.693			
ALP > 135 U/L	0.106	0.704	0.460–1.077			
GGT > 64 U/L	0.115	0.692	0.438–1.094			
PT > 13 s	**0.008**	0.416	0.217–0.794	0.111	1.913	0.861–4.249
AFP > 25 ng/mL	0.695	0.828	0.322–2.128			
CEA > 10 ng/mL	<0.001	0.321	0.201–0.512	**<0.001**	3.398	1.922–6.007
CA199 > 37 U/mL	0.064	0.664	0.430–1.024			
CA125 > 35 U/mL	0.180	0.730	0.461–1.156			
LMR > 3.71	**0.004**	0.489	0.300–0.797	0.110	2.821	1.540–5.166
SII > 730.14	0.973	1.007	0.659–1.541			
NLR ≥ 2.36	**0.024**	0.594	0.378–0.934	0.001	1.577	0.902–2.759
MTD > 5 cm	0.076	1.469	0.960–2.247			
Gender, Male	0.508	0.867	0.567–1.324			
Hepatolithiasis, Yes	<0.001	2.336	1.493–3.655	0.061	1.715	0.975–3.017
HBV carrier, Yes	0.068	1.655	0.964–2.841			
Child-Pugh, B	0.661	0.842	0.391–1.815			
Vascular invasion, Yes	**0.012**	1.770	1.133–2.764	0.072	0.589	0.330–1.048
Local invasion, Yes	**<0.001**	0.291	0.172–0.495	**0.002**	2.946	1.511–5.743
Nerve invasion, Yes	0.007	0.542	0.347–0.847	0.305	1.328	0.772–2.284
Blood transfusion, Yes	**0.005**	0.393	0.203–0.758	0.169	1.740	0.790–3.833
PD, Yes	**<0.001**	0.410	0.265–0.635	**0.001**	2.551	1.495–4.352
TNM staging, III	**<0.001**	0.382	0.247–0.592	**0.014**	2.039	1.154–3.604
Complications Grade, III-IV	0.841	0.953	0.595–1.527			

Bold values means statistically significant.

The univariable and multivariate regression analyses of the risk factors for neoplasm recurrence within 5 years after surgery in all ICC patients are shown in Table 3. Univariate analysis indicated that the variables related to the recurrence of ICC patients are as follows: ALP > 135 U/L, PT > 13 s, CEA > 10 ng/mL, CA199 > 37 U/mL, LMR > 3.71, NLR ≥ 2.36, MTD > 5 cm, combined with hepatolithiasis, vascular invasion, nerve infiltration, blood transfusion, and TNM stage-III. Multivariate analysis suggested that the variables associated with the recurrence of ICC patients are as follows: ALP > 135 U/L, PT > 13 s, CEA > 10 ng/mL, LMR > 3.71, NLR ≥ 2.36, MTD > 5 cm, vascular invasion, blood transfusion, and TNM stage-III.

**Table 3:** Univariable and multivariate regression analyses of risk factors for neoplasm recurrence within 5 years after surgery in ICC patients.

Variate	Univariate analysis			Multivariate analysis		
	*p*- value	HR	95%CI	*p*- value	HR	95%CI
Age > 60 y	0.162	0.740	0.485–1.128			
TBIL>17.1 μmol/L	0.731	0.924	0.587–1.452			
ALB<35 g/L	0.426	1.217	0.750–1.972			
A/G < 1.5	0.335	1.231	0.807–1.878			
ALT>80 U/L	0.645	1.148	0.638–2.066			
AST > 80 U/L	0.555	1.231	0.617–2.457			
ALP > 135 U/L	**<0.001**	2.273	1.477–3.498	**0.048**	1.814	1.006–3.272
GGT > 64 U/L	0.128	1.425	0.903–2.248			
PT > 13 s	0.013	2.280	1.194–4.354	**0.025**	2.587	1.127–5.941
AFP > 25 ng/mL	0.339	1.614	0.605–4.302			
CEA > 10 ng/mL	**<0.001**	5.121	3.116–8.415	**<0.001**	5.506	2.945–10.295
CA199 > 37 U/mL	**0.030**	1.614	1.047–2.488	0.764	0.913	0.504–1.655
CA125 > 35 U/mL	0.161	1.387	0.878–2.190			
LMR > 3.71	**<0.001**	2.642	1.601–4.360	<0.001	4.283	2.250–8.155
SII > 730.14	0.348	0.817	0.535–1.247			
NLR ≥ 2.36	**<0.001**	2.986	1.886–4.730	**0.001**	2.733	1.510–4.948
MTD > 5 cm	**<0.001**	0.403	0.262–0.621	0.029	0.535	0.305–0.939
Gender, Male	0.272	1.267	0.831–1.932			
Hepatolithiasis, Yes	**<0.001**	3.119	1.976–4.926	0.062	1.779	0.305–0.939
HBV carrier, Yes	0.106	0.640	0.373–1.099			
Child-Pugh, B	0.343	1.461	0.667–3.202			
Vascular invasion, Yes	**0.004**	0.521	0.334–0.813	0.003	0.374	0.195–0.717
Local invasion, Yes	0.655	0.901	0.569–1.425			
Nerve invasion, Yes	**0.018**	1.704	1.096–2.649	0.654	0.873	0.483–1.580
Blood transfusion, Yes	**<0.001**	7.391	3.081–17.726	**<0.001**	6.298	2.265–17.513
PD, Yes	0.062	1.497	0.980–2.287			
TNM staging, III	**<0.001**	2.644	1.710–4.089	**<0.001**	3.439	1.857–6.367
Complications Grade, III-IV	0.862	1.042	0.652–1.667			

Bold values means statistically significant.

### Comparison of overall survival and recurrence in HICC and NHICC groups

The comparison of clinical characteristics in the NHICC group and the HICC group is presented in Supplementary Table S1 in the Supporting Information. Among the two cohorts, factors such as ALB, A/G, ALP, GGT, PT, CA199, NLR, MTD, gender, vascular invasion, local invasion, nerve invasion, and blood transfusion between the two groups exhibited a statistically significant difference (*p* < 0.05). Similarly, the ICC patients in the NHICC group and the HICC group were classified into the H-NLR group and the L-NLR group based on the NLR cut-off value of 2.36 for further comparative analysis.

**Figure 4:**
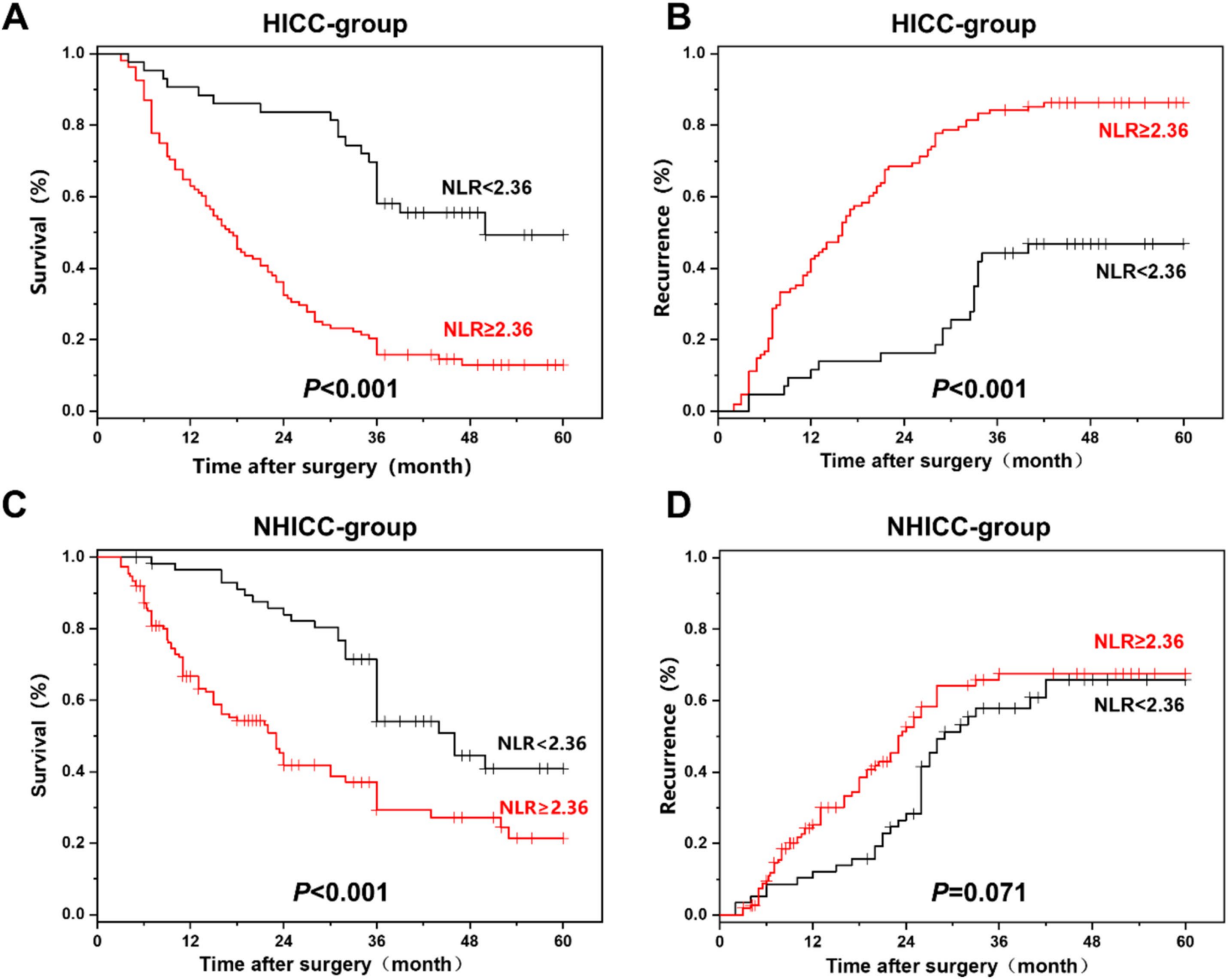
**(A,B)** The survival and recurrence comparison in hepatolithiasis-related intrahepatic cholangiocarcinoma (HICC) patients between the NLR. **(C,D)** The survival and recurrence comparison in non-hepatolithiasis-related intrahepatic cholangiocarcinoma (NHICC) patients between the NLR<2.36 group and the NLR≥2.36 group.

The comparison of overall survival and recurrence in the HICC and NHICC groups between the L-NLR group and the H-NLR group is presented in Figure 4. In the HICC group, the median survival time of 151 HICC patients was 27 months, and the 1-, 3-, and 5-year survival rates were 75.6, 37.9, and 29.4%, respectively. The patients in the L-NLR group had a lower 5-year survival rate than those in the H-NLR group (49.3% vs. 12.9%, *P*<0.001). The median recurrence time was 25 months, and the recurrence rates at 1, 3, and 5 years were 28.1, 65.4, and 71.9%, respectively. The patients in the L-NLR group had a higher 5-year recurrence rate than those in the H-NLR group (46.8% vs. 86.3%, *p* < 0.001). In the NHICC group, the median survival time of 209 NHICC patients was 26 months, and the 1-, 3-, and 5-year survival rates were 74.0, 32.5, and 25.3%, respectively. The patients in the L-NLR group had a lower 5-year survival rate than those in the H-NLR group (40.8% vs. 21.4%, *p* < 0.001). The median recurrence time was 24.5 months, and the recurrence rates at 1, 3, and 5 years were 24.8, 68.8, and 72.5%, respectively. There was no significant difference between the two groups (65.8% vs. 67.6%, *p* = 0.071).

### Analyses of prognostic factors for OS and recurrence in HICC and NHICC groups

The univariable and multivariate regression analyses of the risk factors for OS and recurrence within 5 years after surgery in HICC patients are presented in Tables 4, 5. Univariate and multivariate analyses indicated that the variables associated with the OS of HICC patients are as follows: PT > 13 s, CEA > 10 ng/mL, LMR > 3.71, NLR ≥ 2.36, blood transfusion, and PD; and the variables related to the recurrence of HICC patients are as follows: PT > 13 s, LMR > 3.71, NLR ≥ 2.36, blood transfusion, and PD.

**Table 4:** Univariable and multivariate regression analyses of risk factors for mortality within 5 years after surgery in HICC patients.

Variate	Univariate analysis			Multivariate analysis		
	*p*- value	HR	95%CI	*p*- value	HR	95% CI
Age > 60 y	0.739	1.130	0.551–2.317			
TBIL>17.1 μmol/L	0.260	0.658	0.317–1.364			
ALB<35 g/L	0.778	1.114	0.525–2.363			
A/G < 1.5	0.387	0715	0.335–1.528			
ALT>80 U/L	0.765	0.875	0.364–2.104			
AST > 80 U/L	0.443	0.677	0.250–1.835			
ALP > 135 U/L	0.260	1.521	0.733–3.156			
GGT > 64 U/L	0.398	1.417	0.631–3.179			
PT > 13 s	**0.043**	2.971	1.035–8.525	**0.023**	14.892	1.445–53.498
AFP > 25 ng/mL	0.694	1.562	0.169–14.393			
CEA > 10 ng/mL	**0.015**	2.712	1.214–6.058	**0.030**	4.150	1.150–14.975
CA199 > 37 U/mL	0.482	1.317	0.611–2.835			
CA125 > 35 U/mL	0.233	1.574	0.747–3.314			
LMR > 3.71	**0.006**	3.224	1.409–7.376	**<0.001**	5.340	1.398–7.694
SII > 730.14	0.607	0.829	0.407–1.691			
NLR ≥ 2.36	**<0.001**	7.435	3.351–16.497	**0.001**	7.902	2.601–9.408
MTD > 5 cm	0.202	0.627	0.306–1.284			
Gender, Male	0.445	0.743	0.348–1.590			
HBV carrier, Yes	0.491	0.666	0.210–2.117			
Child-Pugh, B	0.791	1.175	0.357–3.871			
Vascular invasion, Yes	0.909	0.952	0.412–2.203			
Local invasion, Yes	**0.003**	3.934	1.607–9.632	0.286	0.376	0.062–2.271
Nerve invasion, Yes	0.106	1.833	0.880–3.821			
Blood transfusion, Yes	**0.005**	8.587	2.454–40.771	**<0.001**	9.747	3.234–26.848
PD, Yes	**0.019**	2.613	1.169–5.840	**0.024**	5.641	1.261–25.233
TNM staging, III	**0.018**	2.393	1.158–4.945	0.996	1.004	0.272–3.706
Complications Grade, III-IV	0.782	0.893	0.400–1.995			

Bold values means statistically significant.

**Table 5:** Univariable and multivariate regression analyses of risk factors for neoplasm recurrence within 5 years after surgery in HICC patients.

Variate	Univariate analysis			Multivariate analysis		
	*p*- value	HR	95% CI	*p*- value	HR	95% CI
Age > 60 y	0.738	0.882	0.422–1.843			
TBIL>17.1 μmol/L	0.184	0.601	0.824–1.274			
ALB<35 g/L	0.894	1.054	0.486–2.283			
A/G < 1.5	0.270	0.639	0.288–1.417			
ALT>80 U/L	0.832	0.907	0.366–2.249			
AST > 80 U/L	0.281	0.576	0.211–1.570			
ALP > 135 U/L	0.184	1.663	0.785–3.522			
GGT > 64 U/L	0.395	1.434	0.625–3.289			
PT > 13 s	**<0.001**	8.338	3.352–20.740	**0.006**	10.277	1.969–53.644
AFP > 25 ng/mL	0.787	1.358	0.147–12.537			
CEA > 10 ng/mL	**0.004**	3.285	1.459–7.393	0.053	3.324	0.986–11.210
CA199 > 37 U/mL	0.702	1.168	0.526–2.592			
CA125 > 35 U/mL	0.542	1.266	0.594–2.700			
LMR > 3.71	**0.022**	2.651	1.150–6.107	**0.001**	11.916	2.622–24.159
SII > 730.14	0.606	1.214	0.582–2.534			
NLR ≥ 2.36	**<0.001**	7.130	3.171–16.032	**<0.001**	6.688	2.774–21.771
MTD > 5 cm	**0.031**	0.438	0.207–0.926	0.069	0.250	0.056–1.117
Gender, Male	0.535	0.780	0.357–1.708			
HBV carrier, Yes	0.344	0.571	0.179–1.824			
Child-Pugh, B	0.987	1.010	0.305–3.341			
Vascular invasion, Yes	0.932	0.963	0.405–2.291			
Local invasion, Yes	**0.010**	3.270	1.328–8.052	0.142	0.243	0.037–1.603
Nerve invasion, Yes	0.176	1.684	0.791–3.584			
Blood transfusion, Yes	**0.007**	5.924	2.099–20.829	**0.001**	9.851	1.783–30.742
PD, Yes	**0.037**	2.328	1.054–5.140	**0.004**	9.820	2.089–26.162
TNM staging, III	**0.003**	3.127	1.464–6.681	0.532	1.581	0.376–60,650
Complications Grade, III-IV	0.857	1.078	0.478–2.432			

Bold values means statistically significant.

The univariable and multivariate regression analyses of the risk factors for OS and recurrence within 5 years after surgery in NHICC patients are presented in Tables 6, 7. Univariate and multivariate analyses suggested that the variables associated with the OS of HICC patients are as follows: CEA > 10 ng/mL, NLR ≥ 2.36, PD, and TNM stage-III; and the variables related to the recurrence of NHICC patients are as follows: CEA > 10 ng/mL, vascular invasion, and TNM stage-III.

**Table 6:** Univariable and multivariate regression analyses of risk factors for mortality within 5 years after surgery in NHICC patients.

Variate	Univariate analysis			Multivariate analysis		
	*p*- value	HR	95% CI	*p*- value	HR	95% CI
Age > 60 y	0.358	0.775	0.450–1.336			
TBIL>17.1 μmol/L	0.155	0.659	0.372–1.170			
ALB<35 g/L	0.702	0.876	0.445–1.725			
A/G < 1.5	0.652	0.879	0.502–1.539			
ALT>80 U/L	0.774	1.128	0.496–2.565			
AST > 80 U/L	0.338	0.627	0.242–1.629			
ALP > 135 U/L	0.929	1.026	0.589–1.787			
GGT > 64 U/L	0.399	1.279	0.722–2.265			
PT > 13 s	0.763	0.854	0.306–2.385			
AFP > 25 ng/mL	0.554	1.381	0.473–4.030			
CEA > 10 ng/mL	**0.004**	2.149	1.335–4.384	**0.001**	3.315	1.594–6.165
CA199 > 37 U/mL	0.272	1.359	0.786–2.351			
CA125 > 35 U/mL	0.987	1.005	0.543–1.862			
LMR > 3.71	0.568	0.828	0.433–1.583			
SII > 730.14	0.965	1.012	0.585–1.753			
NLR ≥ 2.36	**0.003**	0.414	0.234–0.734	**0.007**	0.396	0.202–0.778
MTD > 5 cm	0.693	0.896	0.520–1.545			
Gender, Male	0.734	1.100	0.634–1.910			
HBV carrier, Yes	0.360	0.744	0.395–1.401			
Child-Pugh, B	0.955	1.031	0.360–2.954			
Vascular invasion, Yes	**0.030**	0.539	0.308–0.941	0.063	0.538	0.280–1.035
Local invasion, Yes	**0.003**	2.838	1.442–5.586	0.201	1.663	0.763–3.624
Nerve invasion, Yes	0.101	1.627	0.910–2.911			
Blood transfusion, Yes	0.625	0.806	0.338–1.918			
PD, Yes	**<0.001**	3.300	1.870–5.823	**0.005**	2.471	1.318–4.632
TNM staging, III	**0.013**	2.016	1.159–3.509	**0.043**	1.962	1.021–3.768
Complications Grade, III-IV	0.637	1.157	0.631–2.121			

Bold values means statistically significant.

**Table 7:** Univariable and multivariate regression analyses of risk factors for neoplasm recurrence within 5 years after surgery in NHICC patients.

Variate	Univariate analysis			Multivariate analysis		
	*p*- value	HR	95% CI	*p*- value	HR	95% CI
Age > 60 y	0.243	0.723	0.420–1.246			
TBIL>17.1 μmol/L	0.405	1.275	0.720–2.258			
ALB<35 g/L	0.864	0.942	0.479–1.855			
A/G < 1.5	0.68	1.125	0.643–1.968			
ALT>80 U/L	0.896	1.056	0.464–2.402			
AST > 80 U/L	0.195	1.905	0.719–5.048			
ALP > 135 U/L	**0.017**	1.985	1.131–3.484	0.220	2.119	0.637–7.046
GGT > 64 U/L	0.534	1.199	0.677–2.125			
PT > 13 s	0.921	0.949	0.342–2.633			
AFP > 25 ng/mL	0.160	2.217	0.731–6.728			
CEA > 10 ng/mL	**0.004**	2.647	1.366–5.131	**0.022**	2.378	1.134–4.983
CA199 > 37 U/mL	0.134	1.521	0.878–2.635			
CA125 > 35 U/mL	0.716	1.121	0.606–2.076			
LMR > 3.71	**0.032**	2.060	1.063–3.993	0.095	1.876	0.896–3.929
SII > 730.14	0.215	0.706	0.407–1.225			
NLR ≥ 2.36	0.148	0.637	0.346–1.174			
MTD > 5 cm	**0.005**	0.453	0.260–0.788	0.054	0.541	0.290–1.011
Gender, Male	0.665	1.129	0.651–1.958			
HBV carrier, Yes	0.729	1.049	0.782–1.456			
Child-Pugh, B	0.774	0.912	0.485–1.715			
Vascular invasion, Yes	**0.017**	0.506	0.288–0.887	**0.006**	0.368	0.180–0.754
Local invasion, Yes	0.113	0.484	0.198–1.186			
Nerve invasion, Yes	0.205	1.451	0.816–2.579			
Blood transfusion, Yes	**0.005**	4.371	1.557–12.270	0.096	2.635	0.843–8.241
PD, Yes	**0.003**	2.314	1.321–4.054	0.218	1.495	0.788–2.834
TNM staging, III	**0.002**	2.415	1.376–40,241	**0.001**	3.278	1.621–6.632
Complications Grade, III-IV	0.925	1.030	0.562–1.887			

Bold values means statistically significant.

### Model built and validation in HICC group

Multivariate analysis and risk score of postoperative survival and neoplasm recurrence in HICC patients 5 years after surgery are presented in Supplementary Tables S2, S3. Multivariate analysis indicated that the variables associated with the OS of HICC patients are as follows: NLR ≥ 2.36 (HR: 4.791, 95%CI: 3.452–10.002), CEA > 10 ng/mL (HR: 3.356, 95%CI: 2.352–9.683), and blood transfusion (HR: 1.332, 95%CI: 0.731–5.017). The prognosis prediction model based on NLR was obtained by adding the total number of points scored in each of the three independent risk factors. The model was: 5-year mortality risk of HICC = 2.120 * NLR + 2.123 * CEA + 3.305 * blood transfusion - 5.292. Multivariate analysis indicated that the variables associated with the neoplasm recurrence of HICC patients are as follows: NLR ≥ 2.36 (HR: 3.321, 95%CI: 1.343–5.174), PT > 13 s (HR: 1.103, 95%CI: 0.231–3.744), blood transfusion (HR: 1.842, 95%CI: 0.892–3.194), and PD (HR: 2.635, 95%CI: 0.877–7.922), and the model was: 5-year recurrence risk of HICC = 3.378 * NLR + 0.072 * PT + 2.472 * blood transfusion +0.969 *PD - 4.630.

**Figure 5:**
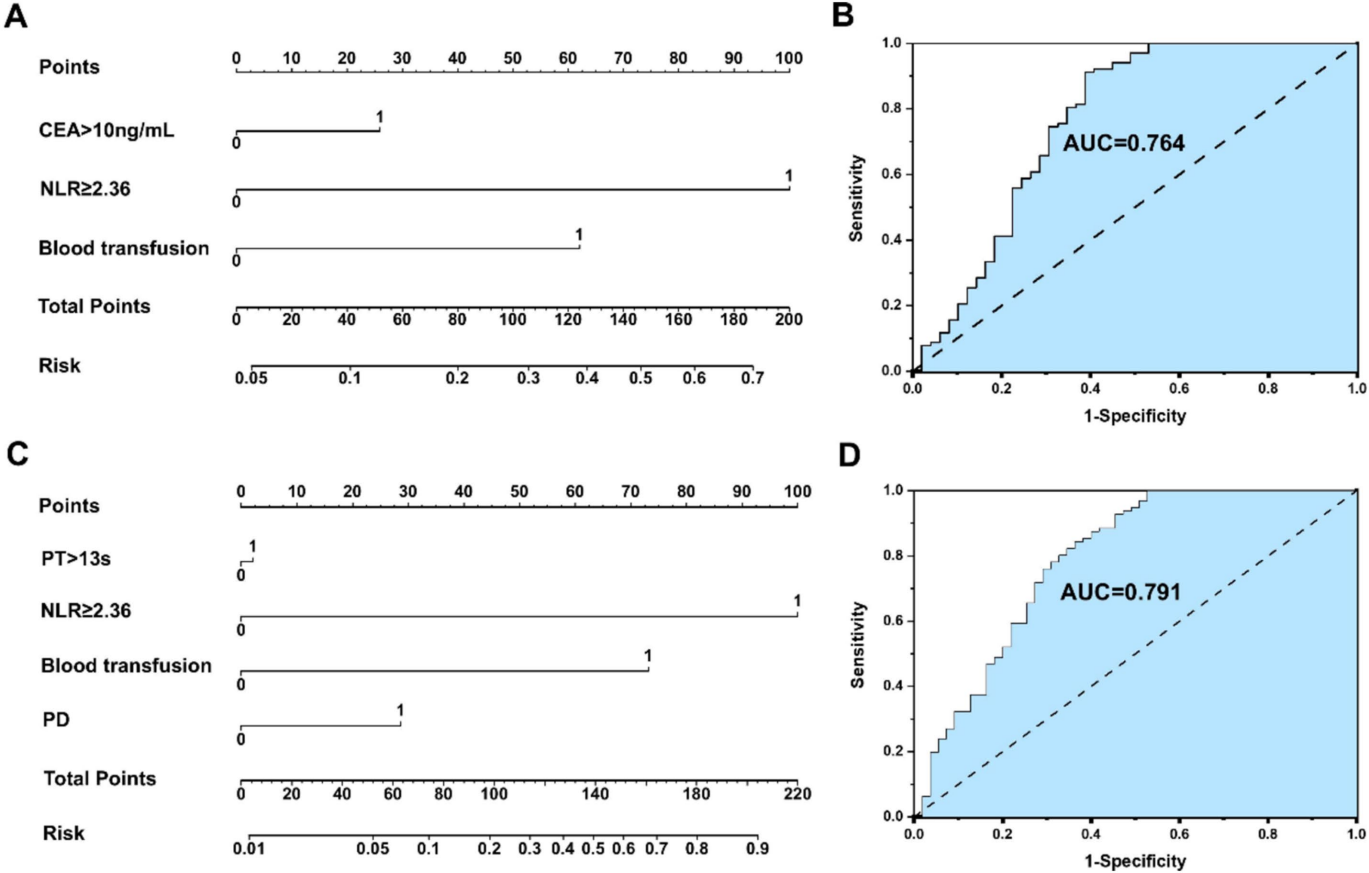
**(A,B)** An NLR-based survival predictive nomogram is established for HICC patients following curative resection and its area under the curve (AUC) in the HICC group. **(C,D)** An NLRbased recurrence predictive nomogram is established for HICC patients following curative resection and its area under the curve (AUC) in the HICC group.

An NLR-based survival/recurrence predictive nomogram is established for HICC patients following curative resection, and its area under the curve (AUC) in the HICC group is shown in Figure 5. It can be seen that NLR shows a higher score in predicting the incidence of 5-year mortality risk of HICC patients, followed by CEA and blood transfusion, and the AUC of the prediction model based on NLR in the HICC group was 0.764, H-L test indicated that *p* value was 0.359 > 0.05 (Figures 5A,B). The NLR also shows a higher score in predicting the incidence of year recurrence risk of HICC patients, followed by blood transfusion, PD, and PT, and the AUC of the prediction model based on NLR in the HICC group was 0.791, H-L test indicated that *p* value was 0.680 > 0.05 (Figures 5C,D).

### Model built and validation in NHICC group

Multivariate analysis and risk score of postoperative survival and neoplasm recurrence in NHICC patients 5 years after surgery are presented in Supplementary Tables S4, S5. Multivariate analysis indicated that the variables associated with the OS of NHICC patients are as follows: NLR ≥ 2.36 (HR: 0.339, 95%CI: 0.177–0.649), CEA > 10 ng/mL (HR: 3.170, 95%CI: 1.617–6.217), and PD (HR: 2.936, 95%CI: 1.063–5.378), and the model was: 5-year mortality risk of NHICC = 1.082*NLR + 1.154*CEA + 1.077*PD - 1.205. Multivariate analysis indicated that the variables associated with the neoplasm recurrence of HICC patients are as follows: NLR ≥ 2.36 (HR: 1.285, 95%CI: 0.665–2.481), CEA > 10 ng/mL (HR: 6.338, 95%CI: 3.151–12.748), vascular invasion (HR: 0.310, 95%CI: 0.155–0.619), and TNM stage-III (HR: 3.214, 95%CI: 1.633–6.326), and the model based on NLR was: 5-year recurrence risk of NHICC = 0.251*NLR + 1.847*CEA + 1.172* vascular invasion +1.168* TNM stage - 1.102.

**Figure 6:**
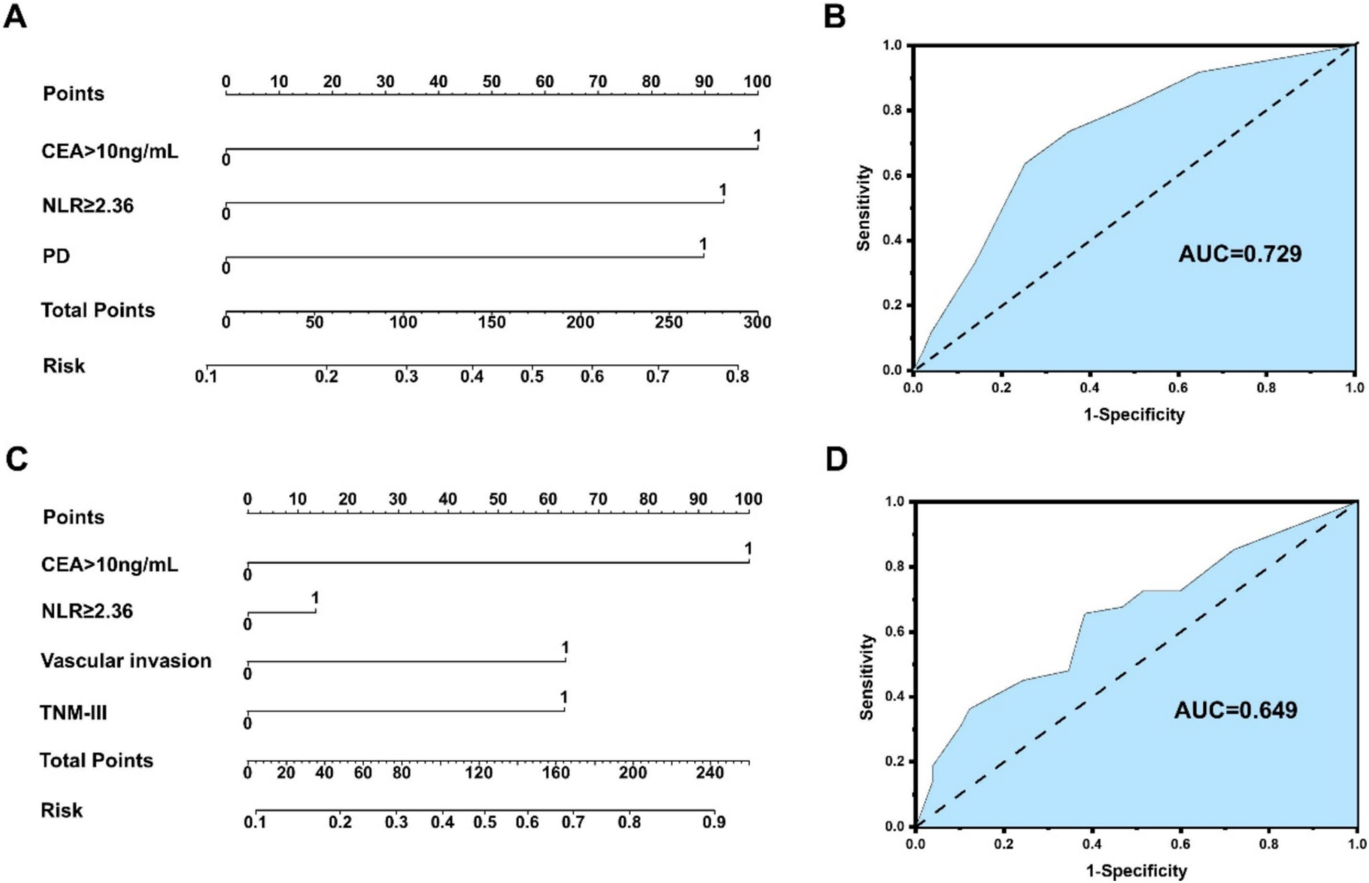
**(A,B)** An NLR-based survival predictive nomogram is established for NHICC patients 34 following curative resection and its area under the curve (AUC) in the NHICC group. **(C,D)** An NLR-based recurrence predictive nomogram is established for NHICC patients following curative resection and its area under the curve (AUC) in the NHICC group.

An NLR-based survival/recurrence predictive nomogram is also established for NHICC patients following curative resection, and its area under the curve (AUC) in the NHICC group is shown in Figure 6. It can be seen that CEA shows a higher score in predicting the incidence of 5-year mortality risk of NHICC patients, followed by NLR and PD, and the AUC of the prediction model based on NLR in the NHICC group was 0.729, H-L test indicated that *p* value was 0.268 > 0.05 (Figures 6A,B). The CEA also shows a higher score in predicting the incidence of year recurrence risk of NHICC patients, followed by vascular invasion and TNM stage, while the NLR did not present a good score in predicting. The AUC of the prediction model based on NLR in the NHICC group was 0.649, H-L test indicated that *p* value was 0.01 < 0.05 (Figures 6C,D).

## Discussion

Surgical resection constitutes an essential approach for the treatment of ICC (24). Surgical indications typically encompass patients presenting with localized lesions, no distant metastasis, and having liver function adequate to withstand surgery (25, 26). Through the collaboration of a comprehensive multidisciplinary team, it has gradually evolved and achieved certain advancements; however, it still confronts challenges, such as the accuracy of preoperative diagnosis, determination of the extent of surgical resection, and the postoperative recurrence rate (27, 28). Postoperative recurrence represents a major clinical issue and is also the focus and difficulty of current treatment. Hence, exploring the high-risk factors of postoperative recurrence and actively adopting corresponding treatments is an important measure to enhance the prognosis of ICC patients.

Serum inflammatory indicators are associated with tumors, and the diagnosis and treatment of ICC is also a research focus of concern, particularly for HICC patients, who are often accompanied by inflammation (29). Thus, serum inflammatory indicators can be utilized to assess the disease status, prognosis, and treatment response of ICC patients. Some studies have reported that certain commonly used serum inflammatory indicators, such as C-reactive protein (CRP), neutrophil/lymphocyte ratio (NLR), systemic immunoinflammatory index (SII), etc. (19, 30, 31), can serve as indicators for tumor diagnosis, prognosis evaluation, and treatment monitoring. However, reports on serum inflammatory indicators of HICC are scarce. There has been no risk factor analysis based on NLR and other clinical indicators in the prognosis of HICC. In this study, three inflammatory indicators, namely LMR, SII, and NLR, were included in the prognostic analysis, and the cut-off values were 3.71, 730.14, and 2.36, respectively. Meanwhile, the ROC curve correlation analysis only indicated that NLR was an independent risk factor influencing the prognosis of ICC patients. Therefore, in this study, NLR was employed as an indicator of serum inflammation in the study of the predictive value of ICC patients after radical surgery.

Studies have shown that a high NLR is associated with a poor prognosis of hepatobiliary tumors (such as liver cancer, gallbladder cancer, etc.), and a high NLR is related to tumor size, invasiveness, and metastatic propensity, indicating a more severe condition of hepatobiliary tumors (32, 33). Some scholars have suggested that NLR can serve as a crucial indicator for the prognosis assessment of hepatobiliary tumors, and the integration of NLR into the prognosis assessment model of hepatobiliary tumors can improve the prediction accuracy (34, 35). In this study, the NLR cut-off value of 2.36 calculated based on the data of this group of samples is more appropriate for the analysis of this sample. According to previous studies, the NLR truncation value typically ranges around 2–3, which is not significantly different from the NLR truncation value set in this study (29). Simultaneously, all patients were divided into NLR < 2.36 and NLR ≥ 2.36 groups. A comparative analysis of clinical data between the groups indicated that the high NLR group had a higher CEA value, more nerve invasion, and a poorer TNM stage. Prognostic analysis suggested that compared with NLR < 2.36, patients in the NLR ≥ 2.36 group had higher 1-, 3-, and 5-year postoperative survival rates and lower 1-, 3-, and 5-year postoperative recurrence rates. Multivariate analysis suggested that the variables associated with the overall survival (OS) of ICC patients are as follows: CEA > 10 ng/mL, NLR ≥ 2.36, local invasion, PD, and TNM stage-III; the variables associated with the recurrence of ICC patients are as follows: ALP > 135 U/L, PT > 13 s, CEA > 10 ng/mL, LMR > 3.71, NLR ≥ 2.36, MTD > 5 cm, vascular invasion, blood transfusion, and TNM stage-III, suggesting that NLR might be correlated with tumor invasion and metastasis.

Studies have indicated that individuals suffering from chronic cholangitis diseases, such as bile duct stones, cholecystitis, cholangitis, etc., have an elevated risk of ICC (36–38). Long-term bile duct disorders may cause chronic inflammation and damage to bile duct epithelial cells, thereby increasing the risk of cancer (39, 40). Some studies have proposed that the relatively favorable prognosis of patients with HICC might be associated with early detection and treatment, as well as the low proportion of HICC in patients with cirrhosis. In contrast, patients with NHICC typically present at a late stage and have a higher postoperative recurrence rate (41, 42). In this study, within the HICC group, the survival rates at 1, 3, and 5 years after surgery in the H-NLR group were higher than those in the L-NLR group. Among patients with NHICC, the survival rates at 1, 3, and 5 years after surgery in the H-NLR group were higher than those in the L-NLR group, and there was no significant difference in the recurrence rate between the two groups. It is suggested that NLR has a poorer ability to predict the prognosis of patients with NHICC compared to those with HICC. Therefore, NLR can serve as a serum inflammatory marker for predicting the prognosis of patients with HICC, and NLR is an important risk factor influencing postoperative survival and recurrence of HICC.

The application of NLR-based tumor prognosis prediction models constitutes an important research domain. The combination of NLR with imaging characteristics, tumor traits, clinicopathological factors, etc., can establish a prognosis prediction model of ICC based on NLR, which enables physicians to assess the prognosis of patients more precisely, thereby formulating individualized treatment plans and follow-up strategies (30, 43, 44). In this study, multivariate regression analyses of multiple variables in the HICC group and the NHICC group were, respectively, conducted, and it was affirmed that the risk factors related to postoperative survival of HICC encompassed: CEA > 10 ng/mL, NLR ≥ 2.36, and blood transfusion; factors associated with postoperative recurrence of HICC: PT > 13 s, NLR ≥ 2.36, blood transfusion, and PD. Risk factors associated with postoperative survival of NHICC included CEA > 10 ng/mL, NLR ≥ 2.36, and PD; factors associated with postoperative recurrence of NHICC were CEA > 10 ng/mL, vascular invasion, and TNM stage-III. The NLR-based survival/recurrence prediction models in the HICC group exhibited excellent predictive capacity (H-L test: 0.359/0.680, AUC: 0.764/0.791); the NLR-based survival prediction model in the NHICC group demonstrated acceptable predictive ability (H-L test: 0.268, AUC: 0.729), while the NLR-based recurrence prediction model did not display an effective predictive ability (H-L test: 0.01, AUC: 0.649), suggesting that the survival and recurrence prediction model constructed based on NLR shows good prediction accuracy and can effectively predict the risk of postoperative adverse prognosis in patients with HICC; however, its predictive value for patients with NHICC is limited.

This study analyzed the prognosis of ICC based on the serum inflammatory index NLR and confirmed that NLR is an independent risk factor influencing the prognosis of HICC. Subsequently, a prognostic model for survival and recurrence after HICC was constructed based on NLR, relevant clinical indicators, pathology, and other factors. This model can predict and analyze the prognosis of HICC patients to a certain extent and contribute to the decision-making and implementation of perioperative comprehensive treatment. However, this study also has certain limitations. As a single-center retrospective study, it lacks multi-center large sample data and has shortcomings such as a small sample size and significant individual differences. In the future, we will undertake a multi-center collaborative research project to jointly conduct a study on the correlation between NLR and the prognosis of ICC patients in multiple regions and centers, thereby increasing the sample size, enhancing the reliability of the research results, and obtaining a more comprehensive understanding of the correlation between NLR and the prognosis of ICC, so as to provide more reliable clinical guidance for the diagnosis, treatment, and prognosis evaluation of this disease.

## Conclusion

NLR is an independent risk factor influencing postoperative survival and recurrence in ICC patients, particularly in HICC patients. Preoperative NLR ≥ 2.36 suggests that patients might have a poor prognosis. For patients with NHICC, the predictive value of CEA may be superior to NLR. The survival and recurrence prediction model constructed based on NLR and other clinical indicators demonstrates good prediction accuracy and can effectively predict the risk of postoperative adverse prognosis in patients with HICC. However, its predictive value for patients with NHICC is limited. This study offers a novel idea for the clinical treatment of HICC patients.

## Data Availability

The datasets presented in this article are not readily available because All the patients encompassed in the current study fulfilled the subsequent criteria: (1) underwent R0 resection, with postoperative pathology confirming ICC; comprehensive clinical case data and follow-up data were accessible; (2) did not undergo tumor-targeted treatments such as transcatheter arterial chemoembolization (TACE), chemotherapy, radiotherapy, targeted therapy, or immunotherapy, etc.; (3) preoperative Child-Pugh score of A/B; (4) had no history of previous malignant tumors. Patients with the following attributes were excluded: (1) R1/2 excision or distant metastasis (M1); (2) postoperative pathology indicating primary liver cancer such as hepatocellular carcinoma, mixed hepatocellular carcinoma, or other types of biliary malignancy; (3) patients with infectious diseases prior to operation; (4) taking hormones, aspirin, clopidogrel, and other drugs influencing peripheral blood cell indicators before surgery; (5) combined with blood diseases or immune system disorders; (6) postoperative perioperative mortality; (7) incomplete clinical data. Requests to access the datasets should be directed to SQ, qishuo@hunnu.edu.cn.be.
