# Supplementary material for: Application of preoperative NLR-based prognostic model in predicting prognosis of intrahepatic cholangiocarcinoma following radical surgery
Source: Front Nutr. 2024 Oct 30;11:1492358. doi: 10.3389/fnut.2024.1492358 (PMC11557473; doi:10.3389/fnut.2024.1492358)
Supplement: Supplementary file 1 [file Table_1.DOCX]

**Supporting Information**

**Application of Preoperative NLR-Based Prognostic Model in Predicting Prognosis of Intrahepatic Cholangiocarcinoma Following Radical Surgery**

Shuo Qi, Zhongzhi Ma, Lian Shen, Jun Wang, Lei Zhou, Bingzhang Tian, Changjun Liu^*^, Kang Chen^*^, Wei Cheng^*^

Department of Hepatobiliary Surgery, Hunan Provincial People's Hospital, The First Affiliated Hospital of Hunan Normal University, Changsha, 410005, Hunan Province, China.

**^*^Corresponding author:**

Wei Cheng, MD., Department of Hepatobiliary Surgery, Hunan Provincial People's Hospital, The First Affiliated Hospital of Hunan Normal University, Changsha, 410005 Hunan Province, China. Email: [chengwei@hunnu.edu.cn](mailto:chengwei@hunnu.edu.cn).

Kang Chen, PhD., Department of Hepatobiliary Surgery, Hunan Provincial People's Hospital, The First Affiliated Hospital of Hunan Normal University, Changsha, 410005 Hunan Province, China. Email: [chenkang@hunnu.edu.cn](mailto:chenkang@hunnu.edu.cn).

Changjun Liu, MD., Department of Hepatobiliary Surgery, Hunan Provincial People's Hospital, The First Affiliated Hospital of Hunan Normal University, Changsha, 410005 Hunan Province, China. Email: liuchangjun@hunnu.edu.cn.

| **Table S1 The comparison of clinical characteristics in NHICC group and HICC group** | | | |  |
| --- | --- | --- | --- | --- |
| **Variate** | **NHICC group**  **(n=209)** | **HICC group**  **(n=151)** | ***P* value** |  |
| Age (y)  TBIL (μmol/L)  ALB (g/L)  A/G  ALT (U/L)  AST (U/L)  ALP (U/L)  GGT (U/L)  PT (s)  AFP (ng/mL)  CEA (ng/mL)  CA199 (U/mL)  CA125 (U/mL)  LMR  SII  NLR  MTD  >5cm  <5cm  Gender  Male  Female  HBV carrier  Yes  No  Child-Pugh  A  B  Vascular invasion  Yes  No  Local invasion  Yes  No  Nerve invasion  Yes  No  Blood transfusion  Yes  No  Poorly differentiation  No  Yes  TNM staging  I、II  III  Complications Grade  I、II  III-V | 58.2±9.7  59.7±32.3  40.6±6.3  1.6±0.4  47.4±43.8  42.5±39.3  167.1±145.0  272.3±188.4  11.0±1.5  41.5±14.2  14.1±7.3  449.3±195.2  83.4±182.7  2.9±1.2  783.6±554.8  3.3±1.6  111（53.1%）  98（46.9%）  122（58.4%）  87（41.6%）  13（6.2 %）  196（93.8%）  194（92.8%）  15（7.2%）  85（40.7%）  124（59.3%）  52（24.9%）  157（75.1%）  71（34.0%）  138（66.0%）  23（11.0%）  186（89.0%）  97（46.4%）  112（53.6%）  93（44.5%）  116（55.5%）  173（82.8%）  36（17.2%） | 59.2±8.7  63.7±31.6  37.8±4.9  1.4±0.3  62.7±54.0  52.4±48.3  240.5±203.5  259.0±246.2  11.3±1.3  49.1±17.1  14.6±9.3  384.3±285.6  72.8±105.3  3.0±1.8  767.6±469.9  4.9±2.7  57（37.7%）  94（62.3%）  54（35.8%）  97（64.2%）  14（9.3%）  137（90.7%）  135（89.4%）  16（10.6%）  35（23.2%）  116（76.8%）  55（36.4%）  96（63.6%）  70（46.4%）  81（53.6%）  35（23.2%）  116（76.8%）  85（56.3%）  66（43.7%）  53（35.1%）  98（64.9%）  125（82.8%）  26（17.2%） | 0.338  0.915  **<0.001**  **<0.001**  0.092  0.255  **<0.001**  **0.044**  **0.048**  0.719  0.186  **0.043**  0.741  0.508  0.812  **0.001**  **0.005**  **<0.001**  0.314  0.260  **0.001**  **0.020**  **0.021**  **0.002**  0.070  0.082  0.558 |  |

| **Table S2 Multivariate analysis and risk score of postoperative survival in HICC patients** | | | | | |
| --- | --- | --- | --- | --- | --- |
| **Variate** | **Multivariate analysis** | | |  | **Regression coefficient** |
|  | ***P* value** | **HR** | **95%CI** | |  |
| NLR≥2.36  CEA>10ng/mL  LMR>3.71  Blood transfusion, Yes  Constant | **<0.001**  **0.001**  0.455  **0.008** | 4.791  3.356  0.674  1.332 | 3.452-10.002  2.352-9.683  0.239-1.898  0.731-5.017 | | **2.120**  **2.123**  0.395  **3.305**  **-5.292** |

| **Table S3 Multivariate analysis and risk score of postoperative neoplasm recurrence in HICC patients** | | | | | | |
| --- | --- | --- | --- | --- | --- | --- |
| **Variate** | **Multivariate analysis** | | | |  | **Regression coefficient** |
|  | ***P* value** | **HR** | **95%CI** | | |  |
| NLR≥2.36  PT>13s  Blood transfusion, Yes  PD, Yes  Constant | **<0.001**  **0.019**  **0.016**  **0.003** | 3.321  1.013  1.842  2.635 | | 1.343-5.174  0.231-3.744  0.892-3.194  0.877-7.922 | | **3.378**  **0.072**  **2.472**  **0.969**  **-4.630** |

| **Table S4 Multivariate analysis and risk score of postoperative survival in NHICC patients** | | | | | |
| --- | --- | --- | --- | --- | --- |
| **Variate** | **Multivariate analysis** | | |  | **Regression coefficient** |
|  | ***P* value** | **HR** | **95%CI** | |  |
| CEA>10ng/mL  NLR≥2.36  PD, Yes  TNM staging, III  Constant | **0.001**  **0.001**  **<0.001**  0.051 | 3.170  0.339  2.936  1.834 | 1.617-6.217  0.177-0.649  1.063-5.378  1.000-3.363 | | **1.154**  **1.082**  **1.077**  0.606  **-1.205** |

| **Table S5 Multivariate analysis and risk score of postoperative neoplasm recurrence in NHICC patients** | | | | | | |
| --- | --- | --- | --- | --- | --- | --- |
| **Variate** | **Multivariate analysis** | | | |  | **Regression coefficient** |
|  | ***P* value** | **HR** | **95%CI** | | |  |
| CEA>10ng/mL  NLR≥2.36  Vascular invasion  TNM staging, III  Constant | **<0.001**  0.456  **0.001**  **0.001** | 6.338  1.285  0.310  3.214 | | 3.151-12.748  0.665-2.481  0.155-0.619  1.633-6.326 | | **1.847**  0.251  **1.172**  **1.168**  **-1.012** |
